# Investigation of the Shadow Effect in Focused Ion Beam Induced Deposition

**DOI:** 10.3390/nano12060905

**Published:** 2022-03-09

**Authors:** Chen Fang, Yan Xing

**Affiliations:** Jiangsu Key Laboratory for Design and Manufacture of Micro-Nano Biomedical Instruments, Department of Mechanical Engineering, Southeast University, Nanjing 211100, China; fangchen@seu.edu.cn

**Keywords:** focused ion beam induced deposition, shadow effect, shadow area, numerical simulation, gas diffusion

## Abstract

Due to the precursor gas flow in the focused ion beam induced deposition process, a shadow effect appears behind the shading structures. This article carries out experiments with phenanthrene as the precursor gas and establishes a numerical model to define the shadow area and estimate the intensity of the shadow effect, considering the morphology of shading structure, the beam shift, and the nozzle parameters. Within the shadow area, the precursor molecule adsorption contribution is estimated by calculating the fraction of precursor gas flow in a specific direction. Finally, the number of precursor molecules within the beam impact area influenced by the shadow effect is obtained, emphasizing the important role of gas surface diffusion. The adsorption contribution within the shadow area differs a lot while deposited structures are similar in height. The error between the simulation and the experimental results is about 5%, verifying the accuracy of the proposed model.

## 1. Introduction

Focused ion beam induced deposition (FIBID) and focused electron beam induced deposition (FEBID) are mature, direct-writing additive technologies at micro/nano scale [1,2,3]. Due to their high resolution, strong local fabrication capability and convenience, they have been widely used in the rapid prototyping of complex structures [4,5,6], the manufacture of functional devices [7,8,9], and the creation of metamaterials [10,11,12]. The dissociation of precursor molecules in FIBID and FEBID process is mainly caused by the secondary electrons [13,14,15]. The growth rate of deposition structure relies on the flux of precursor gas and incident ions or electrons. The flux of incident ions or electrons is controlled by current, voltage and optical lens equipment [16,17]. Many factors affect the precursor gas flux. From the perspective of the dynamic motion of precursor molecules, the precursor gas flux is determined by four items: adsorption, surface diffusion, decomposition, and desorption. The diffusion effect plays an important role in precursor gas replenishment, which can be quantified by gas dynamic theory and extended precursor molecules diffusion model (EPMDM) [18,19,20]. The decomposition and desorption of the precursor gas are determined by the properties of the substrate and precursor gas (such as temperature), and the selected processing parameters [14,18]. Nowadays, most gas injection systems (GIS) are composed of a gas nozzle, and there is a distance and intersection angle between the nozzle and the substrate. Therefore, the precursor gas distribution is not uniform on the substrate surface [20,21,22]. With a different beam shift, the local precursor gas flux varies, affecting the growth rate of the deposition structure; so does the final morphology [20,23]. If the deposition structures are arranged densely, the shadow effect may occur [14,24,25]. Blocked by the shading structure, the precursor gas cannot be injected through GIS to the substrate surface directly within the shadow area; therefore, the precursor gas concentration within the shadow area is at a low level. In the FIBID process, deposition and sputtering coexist. Only if the deposition rate is larger than the sputtering rate, the additive process can successfully be carried out. If a high current with a long residence time is utilized within the shadow area, the dissociation of precursor molecules cannot be compensated, sputtering will overcome deposition, and the additive nanostructures fail to grow.

A perfect shadow effect does not exist in the actual nanofabrication process. Because of the strong directional precursor gas, it can flow along the shading structures edge and reach the shadow area, which makes precursor molecules adsorption possible within the shadow area. This phenomenon also includes the contribution of desorbed and re-adsorbed precursor molecules [13,25]. Besides, surface diffusion of the precursor gas from a high-concentration area into the beam impact area (BIA) further replenishes the precursor gas [26,27]. In nanofabrication, the shadow effect can be avoided by depositing nanostructures towards the precursor gas flow. However, this plan cannot work when the (BIA) is surrounded by symmetric nanostructures, such as a ring with a high ratio. Besides, the shadow effect can also be utilized in the field of glancing angle deposition (GLAD). The shading structures, also called seeds, are constructed beforehand to generate the shadow effect to prevent the precursor molecules adsorption on the substrate surface. The method can make the deposition reactions only happen in the targeted areas, which are not affected by the shadow effect [28,29]. Thus, the range and intensity of the shadow effect need to be investigated and quantified for prevention and application. In our previous study, among densely arranged structures, the secondary effect is observed, which is caused by the secondary particles in the FIBID process [30]. To minimize the secondary effect, phenanthrene is selected as the precursor gas for less scattered ions and the distance between the processing structure and shading structure is larger. Because the shadow area is behind the shading structure, the re-emitted precursor molecules are no longer considered.

In this paper, a numerical model of the shadow effect is proposed based on the extended precursor molecule diffusion model (EPMDM) and the continuous cellular automata (CCA) [15,20]. Considering the nozzle parameters, the morphology of the shading structure, and beam shift, the contour of the shadow area on the XOY plane is determined. Besides, the boundary height of deposition structure within the shadow area is calculated in the z direction, and the 3D shadow effect range is defined. Within the shadow area, the amount of flowing precursor gas is estimated for adsorption based on a probability model. Afterward, considering the surface diffusion, the precursor gas concentration within the BIA under equilibrium conditions is estimated and the final deposition morphology is captured.

## 2. Materials and Methods

### 2.1. Experimental Materials and Methods

All experiments are carried out on an FEI Helios G4 dual-beam system. The nozzle parameters are fixed, with a tilted angle of 60°, a height of 160 μm, and an inner diameter of 600 μm. Assisted with phenanthrene as the precursor gas, all shading structures are deposited by 16 keV, 0.75 nA Ga ions using a raster scan. The choice of the lower voltage and higher current is to accelerate the additive process. The nanowalls in Section 3.1.1 are constructed for 106 and 198 s to reach the height of 2.5 and 4.3 μm, respectively. The fabrication time of the nanowall in Section 3.1.2 is 215 s. The fabrication time of the circular, rectangular, and diamond shading in Section 3.1.3 is 120, 140, and 135 s, respectively. The overlap is set as 30%. The pillars are fabricated by 30 keV, 90 pA Ga ions in spot mode, which refers to a continuous beam illumination at a given point. The nozzle shift is the same as the beam shift in the experiments because the distance between the shading structure and the beam impact site is much smaller than that between the nozzle and the shading structure. In Section 3.1.1, all pillars are deposited for 60 s. In Section 3.1.2, the total processing time for each pillar is 6 min. The fabrication process is divided into 6 parts, and each part takes 1 min. The two pillars are deposited alternately every minute to minimize the height difference between them for preventing the shadow effect between pillars. In Section 3.1.3 the pillars are deposited for 120 s. The pressure in the chamber increases from 9.59 × 10^−5^ Pa to 7.8 × 10^−4^ Pa when the precursor gas is injected. The silicon substrate is conducted with surface treatment as follows: First, the silicon substrate is single-side polished. Second, the silicon substrate is soaked for 15 min in acetone. Third, in the acetone circumstance, ultrasonic cleaning is conducted for 15 min. Fourth, the silicon substrate is cleaned with distilled water for 2 min. Finally, the silicon substrate is dried in a drying oven for 30 min.

### 2.2. Simulation Methods

**Figure 1:**
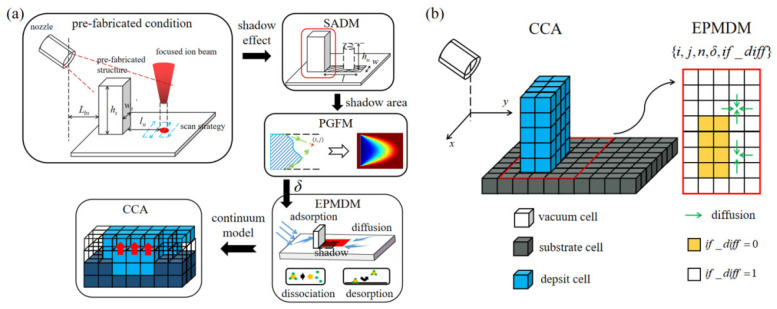
(**a**) The workflow of SADM, PGFM, EPMDM, and CCA; (**b**) schematic diagram of the CCA and EPMDM.

The numerical model consists of four parts, the shadow area defining model (SADM), the precursor gas flow model (PGFM), the EPMDM, and the CCA. The workflow of the whole numerical simulation model is demonstrated in Figure 1a. The SADM is established to define the shadow area behind the shading structure. The PGFM is to calculate the precursor gas adsorption contribution within the defined shadow area. The EPMDM is utilized to estimate the number of precursor molecules and the growth rate. Finally, the capture of deposition structure contour is completed in the CCA.

#### 2.2.1. The CCA Model

In the CCA, three different cells are defined to represent three conditions: vacuum, substrate, and deposit. The cellular space contains the substrate surface and the vacuum space above. We introduce a threshold value *ω* for cell species transfer. The cell occupation ρ is refreshed at the end of a time step. If *ρ* of a vacuum cell reaches *ω*, it turns into a deposit cell, and *ω* is reset to zero.

#### 2.2.2. The EPMDM Model

The EPMDM is established to quantify the number of precursor molecules by solving the equation in discrete cells:

(1) $$\frac{\partial n}{\partial t}=SJ(1-\frac{n}{n_{0}})+D(\frac{\partial^{2}n}{\partial x^{2}}+\frac{\partial^{2}n}{\partial y^{2}})-\sigma fn-\frac{n}{\tau },$$

where *S* is the adhesion probability, *J* is the local precursor gas flux, *n*_0_ is the number of precursor gas molecules at saturation within a cell, *n* is the number of precursor gas molecules within a cell, *D* is the diffusion coefficient, *σ* is the reaction cross section, *f* is the number of incident ions, and *τ* is the residence time. Two additional elements are added into the property of a single cell in the EPMDM: (1) *δ* value to record ratio of precursor gas flow between regions with and without shadow effect; (2) *if_diff* value, which is a Boolean value, is used to determine the participation of cell in gas surface diffusion. The *if_diff* value of the cell occupied by the shading structure is set as 0, thus, in the subsequent calculation of the surface diffusion contribution, these cells will no longer be considered. Meanwhile, the contribution of the precursor gas adsorption is:

(2) $$Com_{flux}(i,j)=S\cdot \delta \cdot J\cdot (1-\frac{n(i,j)}{n_{0}}),$$

where *n*(*i*, *j*) is the number of precursor gas within the cell at (*i*, *j*). The calculation of the surface diffusion contribution is:

(3) $$Com_{diff}(i,j)=\frac{D}{4}\cdot (M_{if\_diff}\cdot M_{neighbour}-n(i,j)),$$

where, *M_if_diff_* is a 1 × 4 matrix as: [*if_diff*(*i* − 1, *j*), *if_diff*(*i* + 1, *j*), *if_diff*(*i*, *j* − 1), *if_diff*(*i*, *j* + 1)], *M_neighbour_* is a 4 × 1 matrix as [*n*(*i* − 1, *j*), *n*(*i* + 1, *j*), *n*(*i*, *j* − 1), *n*(*i*, *j* + 1)]^T^. The depletion of precursor gas can be described by

(4) $$\left\{ \begin{array}{l} Dep_{diss}(i,j)=\sigma \cdot f(i,j)\cdot n(i,j)\\ Dep_{des}(i,j)=\frac{n(i,j)}{\tau } \end{array}\right..$$

The schematic diagram of the EPMDM is shown in Figure 1b. The surface diffusion coefficient *D* is taken as 1.25 μm^2^ s^−1^, and the residence time of precursor molecule *τ* is set as 0.5 s in the simulation [30].

#### 2.2.3. The Shadow Area Defining Model

**Figure 2:**
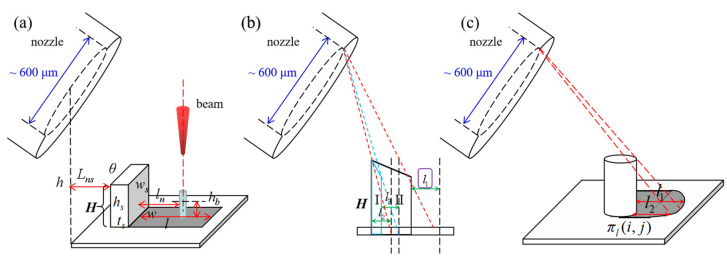
The schematic diagram of the shadow area defining model. (**a**) The definition of elements in the SADM; (**b**) the calculation of the shading length when there are different structure heights under the same *i* value; (**c**) the influence of the contour of the shading structure on the shading area.

The shadow area defining model (SADM) is proposed to define the shadow area on the XOY plane, and to calculate the boundary height *h_b_*(*i*, *j*) of the processing structure grown within the shadow area in the *z* direction. The shadow area consists of a series of shading lengths *l*(*i*, *j*), which depend on the nozzle height *h*, nozzle shift *L_ns_*, and the structure height matrix *H*, as shown in Figure 2a. The length, width, and height of the shading structures are denoted with *w_s_*, *t_s_*, and *h_s_*. The angle between the nozzle and the shading structure can be calculated as:

(5) $$\theta =\arctan(\frac{h}{L_{ns}}),$$

and the shading length can be calculated:

(6) $$l(i,j)=\frac{H(i,j)}{\tan(\theta )}.$$

There may be multiple corresponding structural heights with the same *i* value, as shown in Figure 2b. It is necessary to calculate the shading length corresponding to each height and the largest value is taken. The contour of the shading structure will affect the shadow area. In Figure 2c, where the shading structure is a cylinder, the shadow area presents a circular feature, different from the rectangular feature in Figure 2a.

The boundary height *h_b_*(*i*, *j*) is a critical value. If the processing structure grows higher than the boundary height, the shadow effect will disappear. The boundary height depends on angle *θ*, the distance from the shading structure *l_n_*, and the shading length *l*, which can be expressed as:

(7) $$h_{b}(i,j)=\left\{ \begin{array}{l} \tan(\theta )(l-l_{n})\;\;\;\;\;\;\;(l>l_{n})\;\\ 0\;\;\;\;\;\;\;\;\;\;\;\;\;\;\;\;\;\;\;\;\;\;\;\;\;\;\;\;(l<l_{n}). \end{array}\right.$$

When *l* < *l_n_*, *h_b_* remains as 0. Under this situation, although the shading structure still produces the shadow effect, the shadow area does not cover the BIA, so the growth process is not affected by the shadow effect. The interactions among the elements in Figure 2a are exhibited in Figure S1 to provide an intuitive demonstration of where the shadow effect arises.

#### 2.2.4. The Precursor Gas Flow Model

Within the shadow area, the precursor gas can flow along the shading structure edges and reach the shadow area to compensate for the precursor gas consumption. The precursor gas flow model (PGFM) is established to calculate the ratio *δ*. The model is based on the following assumptions: (1) only the precursor gas near the shading structure is considered, and the influence of the precursor gas away from it is reflected in the background pressure *P_b_* [22]; (2) the collision and recoil between precursor gas molecules and the substrate are neglected; (3) after passing the shading structures, precursor gas flow direction will follow a cosine distribution; (4) the boundary condition of the model is: *δ* = 0 along the edge of the shading structure, and achieves unity at the contour of the shadow area.

**Figure 3:**
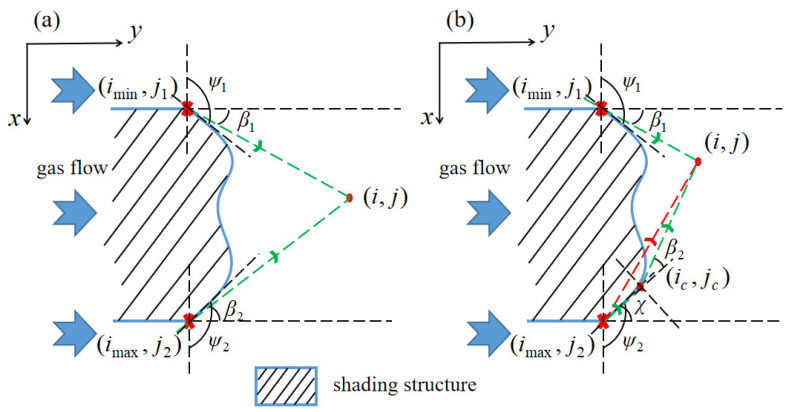
The schematic diagram of the PGFM. (**a**) The precursor gas flow can directly reach the cell (*i*, *j*) after passing through the cell (*i_min_*, *j*_1_) and the cell (*i_max_*, *j*_2_); (**b**) the precursor gas flow reaches the cell (*i*, *j*) after passing through the cell (*i_c_*, *j_c_*) and (*i_min_*, *j*_1_).

For the cell (*i*, *j*), there are two sources of precursor gas adsorption. One reaches the cell after passing the cell (*i_min_*, *j*_1_) with a flowing angle *β*_1_, as shown in Figure 3a. The other one passes the cell (*i_max_*, *j*_2_) with a flowing angle *β*_2_, or after flowing along the shading structure, it passes through the cell (*i_c_*, *j_c_*) and finally reaches the cell (*i*, *j*), as shown in Figure 3b. The flowing angle *β*_1_ and *β*_2_ in Figure 3a are calculated as:

(8) $$\left\{ \begin{array}{l} \beta_{1}=\arctan(\frac{i-i_{min}}{j-j_{1}})\\ \beta_{2}=\arctan(\frac{i_{max}-i}{j-j_{2}}) \end{array}\right..$$

Meanwhile, the sum of *δ* in all the flowing directions should be united. After passing the cell (*i_min_*, *j*_1_), the range of the flow direction is [*π* − *ψ*_1_, *π*], where *ψ*_1_ is the tangent angle of shading structure contour at (*i_min_*, *j*_1_). Similarly, after passing the cell (*i_max_*, *j*_2_), the flow direction range is [0, *ψ*_2_], where *ψ*_2_ is the tangent angle at (*i_max_*, *j*_2_). Then *δ* at the cell (*i*, *j*) can be expressed as:

(9) $$\delta (i,j)=k_{1}\cos^{q}(\beta_{1})+k_{2}\cos^{q}(\beta_{2}),$$

where *k*_1_ and *k*_2_ are adaptation parameters, which are related to the exponent *q* and satisfy the probability restriction as follows:

(10) $$\left\{ \begin{array}{l} \int_{\pi -\psi_{1}}^{\pi }\;k_{1}\cos^{q}(\zeta )d\zeta =1\\ \int_{0}^{\psi_{2}}\;k_{2}\cos^{q}(\zeta )d\zeta =1. \end{array}\right.$$

The value of *q* is larger than 1, and is used to calibrate the experimental results. When precursor gas flow cannot directly reach the target cell (*i*, *j*) through the cell (*i_min_*, *j*_1_) or the cell (*i_max_*, *j*_2_), as shown in the red dashed line in Figure 3b, the flow path needs to be divided into a two-stage calculation. Firstly, the precursor gas flow passes through the cell (*i_max_*, *j*_2_) and reaches the tangent point cell (*i_c_*, *j_c_*); Secondly, the precursor gas flow passes through the tangent point cell (*i_c_*, *j_c_*), and finally reaches the cell (*i*, *j*). The flowing angle in the first stage *χ* is calculated as:

(11) $$\chi =\arctan(\frac{i_{max}-i_{c}}{j_{c}-j_{2}}),$$

meanwhile, the sum of the probability in all flow directions is united:

(12) $$\int_{0}^{\psi_{2}}k_{3}\cos^{q}(\xi )d\xi =1.$$

The flowing angle *β*_2_ of the second stage can be calculated as:

(13) $$\beta_{2}=\arctan(\frac{i_{c}-i}{j-j_{c}})-\chi ,$$

with its constraint condition:

(14) $$\int_{\chi }^{\frac{\pi }{2}+\beta_{2}+\chi }k_{4}\cos^{q}(\zeta )d\zeta =1.$$

Therefore, *δ* at the cell (*i*, *j*) is:

(15) $$\delta (i,j)=k_{1}\cos^{q}(\beta_{1})+k_{3}\cdot k_{4}\cos^{q}(\chi )\cos^{q}(\beta_{2}).$$

## 3. Results and Discussion

### 3.1. Experimental Results

#### 3.1.1. Pillars with Different Distances from the Shading Structure

**Figure 4:**
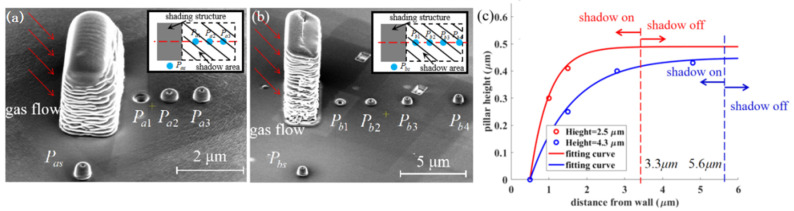
Pillars grown behind an (**a**) 1.8 μm × 3.8 μm × 2.5 μm; (**b**) 1.8 μm × 3.8 μm × 4.2 μm rectangular shading structure; (**c**) experimental results and fitting curve of the pillar heights. The nozzle is on the left of the figure.

Rectangular shading structures with a size of *t_s_* = 1.8 μm, *w_s_* = 3.8 μm, *h_s_* = 2.5 μm and *t_s_* = 1.8 μm, *w_s_* = 3.8 μm, *h_s_* = 4.3 μm are manufactured. The nozzle shift *L_ns_* = 210 μm. Pillars are deposited on the midline of the shading structures, with different distances from the structure, in spot mode, as shown in Figure 4a,b. Meanwhile, pillars *P_as_* and *P_bs_* are fabricated outside the shadow area as standard. The nozzle is located on the left in Figure 4. The heights of pillars grown within the shadow area are gradually saturated to the standard height with a larger distance from the shading structures, while the widths remain constant, indicating that the shadow effect gradually disappears. The detail of the pillars is recorded in Table S1. With a distance from the shading structures of *l_n_* = 3.3 μm and 5.6 μm, the heights of the deposited pillars (*P_a_*_3_, *P_b_*_4_) are very close to the standard. Figure 4c presents the fitting curve of the pillar heights. The shadow effect disappears at 3.3 μm and 5.6 μm, respectively, corresponding to the shading structure height of *h_s_* = 2.5 μm and 4.3 μm. The holes in the nanopillars are caused by sputtering.

#### 3.1.2. Time-Dependent Variation in Heights and Growth Rates of Pillars Grown within the Shadow Area

**Figure 5:**
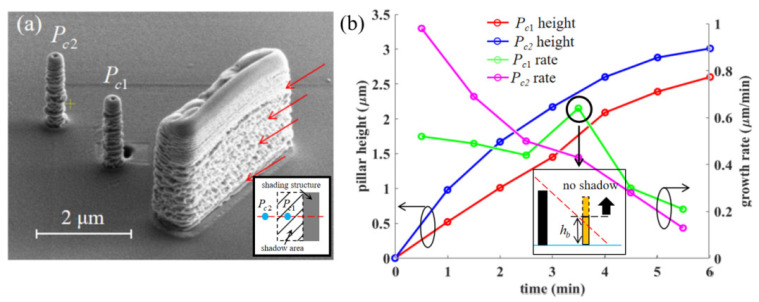
(**a**) The experimental results of pillars grown at 2.5 μm and 5 μm behind the shading structure with a size of 1.7 μm × 6.5 μm × 4.0 μm; (**b**) the time-dependent height and growth rate curves of the pillars. The red arrows indicate the precursor gas flow.

A rectangular shading structure of *t_s_* = 1.7 μm, *w_s_* = 6.5 μm, *h_s_* = 4.0 μm is manufactured with a nozzle shift *L_ns_* = 180 μm. Two pillars are deposited at the midline, 2.5 μm and 5 μm away from the shading structure. The result is shown in Figure 5a. The heights of *P_c_*_1_ and *P_c_*_2_ are 2.6 μm and 3.0 μm. Figure 5b shows the time-dependent variation of the heights and growth rates. As the height gradually increases, the growth rate of the *P_c_*_2_ decreases, attributed to the weakening of the surface diffusion and thermal effect [2,15]. The average growth rate of *P_c_*_2_ is 8.33 nm/s, which is slightly greater than the growth rate of the pillars in experiment 3.1.1. This is explained by the increase in precursor gas flux due to a smaller nozzle shift, thus the precursor gas concentration within the BIA climbs up. The growth rate of the *P_c_*_1_ possesses the same downward trend as the *P_c_*_2_. However, when the processing time is between 3 min and 4 min (corresponding to the structure height between 1.6 μm and 1.9 μm), there is a sudden increase. It is believed that this phenomenon arises because of the boundary height. After exceeding this height, the shadow effect will disappear. For *P_c_*_1_, the boundary height *h_b_* is between 1.6 μm and 1.9 μm, while there is not any sudden variation of growth rate during the fabrication of *P_c_*_2_, indicating that the position is 5 μm away from the shading structure is already outside the shadow area.

#### 3.1.3. The Influence of the Shading Structure’s Morphology on the Shadow Effect

**Figure 6:**
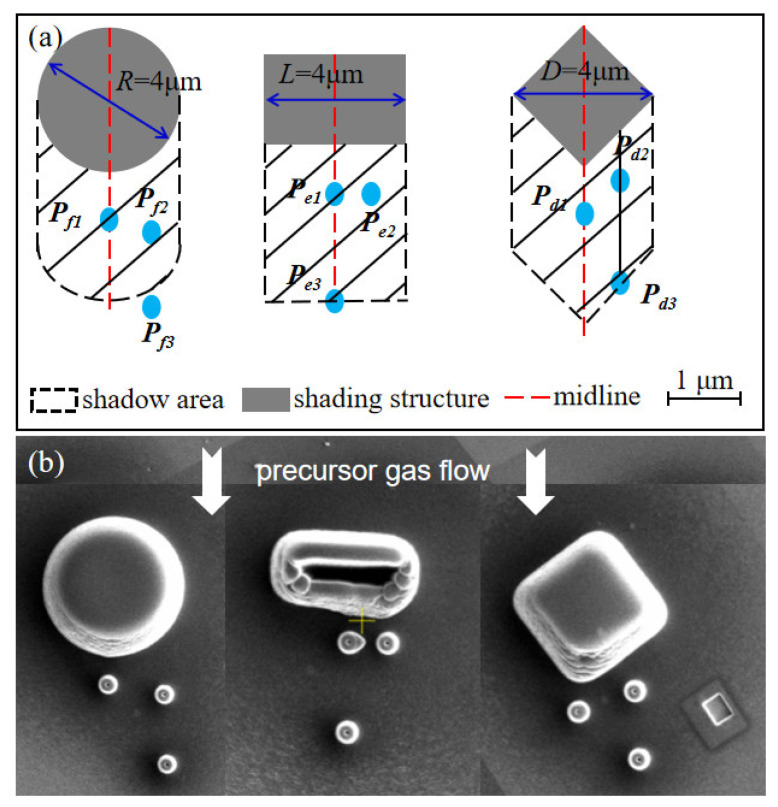
The (**a**) schematic, and (**b**) experimental results of three shading structures with different morphologies (circular, rectangular, diamond). The precursor gas flow direction is from the top to bottom.

**Figure 7:**
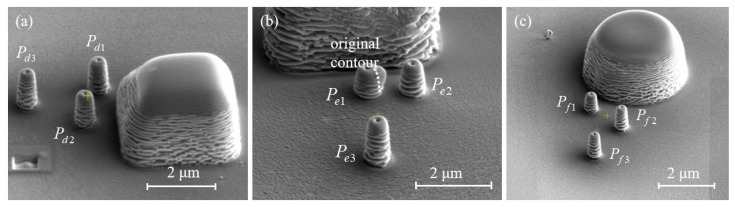
(**a**) Pillars *P_d_*_1_, *P_d_*_2_, and *P_d_*_3_ grown behind the diamond shading structure; (**b**) pillars *P_e_*_1_, *P_e_*_2_, *P_e_*_3_ grown behind the rectangular shading structure; (**c**) pillars *P_f_*_1_, *P_f_*_2_, *P_f_*_3_ grown behind the circular shading structure.

With a nozzle shift of 300 μm, circular, rectangular, and diamond shading structures are fabricated, as shown in Figure 6. The precursor gas flow direction is from the top to bottom. The diameter of the circular shading structure is 4 μm (*w_s_* = 4 μm) and the height *h_s_* = 2.9 μm. The rectangular shading structure has *w_s_* = 4 μm, *t_s_* = 1 μm, *h_s_* = 2.9 μm. The diagonal length of the diamond shading structure is 4 μm with the same side length (*w_s_* = 4 μm), and the height *h_s_* is also 2.9 μm. To avoid the secondary effect (See Figure S3), behind the shading structures, the pillars *P_d_*_1_, *P_e_*_1_, and *P_f_*_1_ are deposited on the midline of the structures all at a position 1 μm from them while the pillar *P_e_*_3_ sits on the midline with a position of 4 μm. The pillars P*_d_*_2_, and *P_e_*_2_ are deposited at a position shifting from the midline by 1 μm and 1 μm away from the structures. The pillar *P_f_*_2_ is deposited at a position shifting from the midline by 1 μm and 2 μm away from the structures. The pillars *P_d_*_3_ and *P_f_*_3_ are deposited at a position shifting from the midline by 1 μm and 4 μm away from the structures. The layout of the pillars is illustrated with a higher resolution in Figure 7. All the pillars adopt the spot mode, and the total processing time is 2 min. The size of the pillars deposited is relatively small compared with that of the shading structures and the distance between them, thus the shadow effect imposed by prefabricated pillars can be neglected. The pillars in the experiments vary in heights but slightly change in widths. The pillars *P_d_*_1_, *P_e_*_1_, and *P_f_*_1_ are the shortest among their groups, indicating that the shadow effect is more prominent. As the BIA shifts from the midline, the pillars *P_d_*_2_, *P_e_*_2_, and *P_f_*_2_ get higher. The heights of *P_d_*_3_ and *P_f_*_3_ are 1.46 μm and 1.48 μm, respectively, which is very close, indicating that the shadow effect almost disappears. The height of the *P_e_*_3_, which is also 4 μm away from the shading structure, is 1.38 μm, explaining that the shadow effect gradually weakens as the distance from the midline increases. After passing the shading structures, the probability of precursor gas flow directions to the midline is smaller than that to other positions. At the shifted position, the pillar grows higher with a larger distance from the shading structures. Therefore, the intensity of the shadow effect follows a radial shape. The center of the radial shape sits at the midline of shading structures, clinging to the structure surface. It is noted that the shadow effect behind the rectangular shading structure is more obvious because the pillar heights are shorter than those behind diamond and circular shading structures. Compared with the rectangular shading structure, the precursor gas can flow along the contours of the diamond and circular shading structures to reach the shadow area. Moreover, the flow direction of precursor gas is distributed within a range of 180° after passing the rectangular shading structure, while for the diamond and circular shading structures, this range will be reduced. Consistent with the PGFM, *δ* within the BIA increases. For the circular shading structure, the shadow effect vanishes rapidly, which confirms that the shadow effect imposed by the pillars is negligible. Through an energy-dispersive X-ray spectrometer (EDS), we found that the shadow effect also influences the content of the deposit materials. The C content increases with a weaker shadow effect and the Ga content decreases. The details of the pillars are exhibited in Figure S2. It is noted that in Figure 7b, *P_e_*_1_ is deformed due to the secondary effect [30]. To further validate our findings, we also fabricated pillar arrays behind the shading structures. The experimental results and discussions can be found in Figure S3.

### 3.2. Simulation Results

The simulation program is compiled by C++, the CPU of the computer is Intel(R) Core(TM) i5-2400, with 8G RAM. The cell size of the CCA is 40 nm × 40 nm × 40 nm, and the cell size of the EPMDM is 40 nm × 40 nm. The length of the C-C bond in precursor gas phenanthrene is 0.14 nm, and the molecular area of phenanthrene is about 5.1 × 10^−2^ nm*^2^*. Therefore, in the EPMDM, the maximum number of precursor molecules in each cell is *n*_0_ = 10,000. The selection of processing parameters in the simulation is consistent with the experiments. The flow chart of the simulation is as follows: Firstly, the range of the shadow effect is decided by SAMD. Then, within the shadow area, the PGFM is initiated to generate the *δ* distribution. Afterward, in EPMDM, the contribution of adsorption, surface diffusion, dissociation, and desorption are calculated to obtain the number of precursor molecules. The growth rate is determined by the incident ions and precursor molecules, which are taken into the CCA as the evolution rate. The dynamic contour of the processing structure is captured [20].

#### 3.2.1. Calculation of the Shading Length and Boundary Height

In experiment 3.1.1, the nozzle shift *L_ns_* is 210 μm. According to the SADM, the angle *θ* between the nozzle and the shading structure is 37°. Because of a rectangular shading structure, the shading length is constant. The simulated shading lengths, *l* = 3.3 μm and 5.6 μm, are consistent with the experimental shading lengths of 3.4 μm and 5.6 μm. In experiment 3.1.2, the nozzle shift *L_ns_* is 180 μm, and the angle *θ* between the nozzle and the shading structure is 42°, thus the simulated shading length is 4.5 μm. The distance between the pillar *P_c_*_2_ and the shading structure is 5 μm, which exceeds the shading length, so *P_c_*_2_ is not affected by the shadow effect, and its boundary height *h_b_* = 0 μm. The distance between the pillar *P_c_*_1_ and the shading structure is 2.5 μm, meaning that *P_c_*_1_ is affected by the shadow effect, and its boundary height is 1.8 μm, which is consistent with the height of 1.6 μm~1.9 μm found in the experiment where a sudden change in rate is observed.

#### 3.2.2. Calculation of the δ Distribution within the Shadow Area

**Figure 8:**
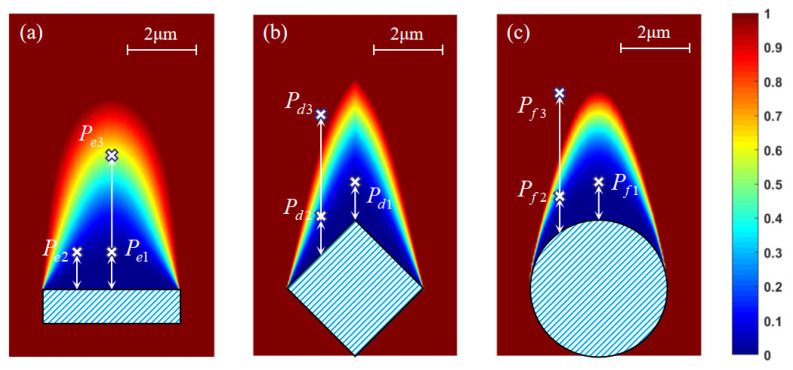
The *δ* distribution behind (**a**) the rectangular shading structure; (**b**) the diamond shading structure (**c**) the circular shading structure calculated from the PGFM. The precursor gas flows from the bottom to the top.

**Figure 9:**
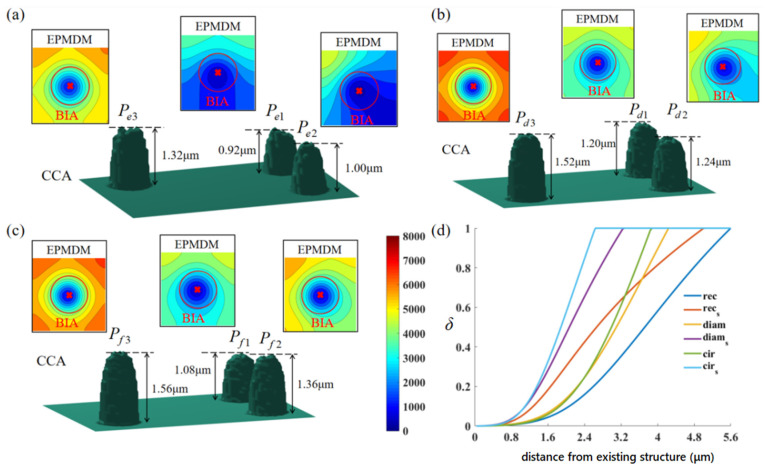
The simulation results of the EPMDM and CCA with separated groups of pillars. (**a**) Behind the rectangular shading structure; (**b**) behind the diamond shading structure; (**c**) behind the circular shading structure; (**d**) the variation trend of the adsorption probability of precursor molecules at different positions.

In the PGFM, the exponent needs to be firstly decided, and *k_i_* can be determined by the probability constraint. Therefore, trial simulations are conducted to find the most suitable parameters (See Figure S4). Taking the experimental conditions into the PGFM, the *δ* distribution in the experiments can be obtained. Behind the shading structures with three different morphologies (rectangular, diamond, circular), the calculated results by the PGFM are shown in Figure 8. The precursor gas flows from the bottom to the top. The height of the shading structure is 2.9 μm, so the shading length l is 5.4 μm. The nine pillars (*P_e_*_1_~*P_f_*_3_) are marked in Figure 8. Compared with the rectangular shading structure, the shadow area behind the diamond and circular shading structures shrink, and *δ* within the shadow area rapidly recovers, especially at the edge of the shading structure. Figure 9d presents the variation trend of *δ* on the midline of the shading structure (rec, diam, cir) and the position shifting from the midline by 2 μm (rec*_s_*, diam*_s_*, cir*_s_*). In accordance with the experimental results, the shadow effect of the rectangular shading structure is the most prominent. Whatever the shifting distance, the shadow effect disappears until the BIA position moves away from the rectangular shading structure about 5 μm. However, for diamond and circular shading structures, *δ* rises sharply as the distance from the shading structure increases. On the midline, the shadow effect behind the diamond and circular shading structures disappears at a distance of 4 μm, and the distance becomes 3 μm when shifting from the midline. The shadow effect behind the diamond shading structure is slightly stronger than that behind the circular shading structure.

#### 3.2.3. Calculation of Precursor Gas Concentration and Pillar Height

The shading structures are constructed artificially, consistent with the size in the experiments, thus they have an ideal and smooth contour. The deposited pillars are totally stimulated, resulting in a rough surface (See Figure S5). According to the PGFM, the lowest *δ* is 0.02 (*P_e_*_1_), and the highest is 1 (*P_d_*_3_, *P_f_*_3_). However, in the experiments, the difference between the heights of the pillars deposited at these positions is not very large. The height of *P_e_*_1_ is 0.89 μm, and that of *P_f_*_3_ is 1.48 μm. *δ* differs by two orders of magnitude, but the height differs by only about 40%. This is attributed to two reasons. Firstly, when the pillar *P_f_*_3_ grows, the reaction is in an ion-limited state. Secondly, the surface diffusion effect becomes more prominent when the precursor gas concentration within the BIA is lower. As a result, the amount of precursor gas supplemented by the surface diffusion effect during the growth of *P_e_*_1_ is much more than that during the growth of *P_f_*_3_. It is proved by utilizing the EPMDM to calculate the precursor gas concentration within the BIA, as shown in Figure 9 with the separated groups of pillars.

Although *δ* varies greatly, resulting in a large difference between the precursor gas adsorption, the number of precursor molecules within the BIA all ranges from 1000 to 2000 due to the surface diffusion, which explains the similarity between the pillar heights. Based on the continuum model [14], the CCA can simulate the growth of the pillar and capture the final contour. The simulation heights of each pillar are listed in Figure S6. The error between the simulation and the experimental results is about 5%.

## 4. Conclusions

This paper studies the shadow effect in FIBID from experiments with phenanthrene as precursor gas and numerical simulation. The experimental results show that the shadow effect features a spatial range dependent on the geometric parameters of the shading structures. Meanwhile, the C content in the deposition structure gradually climbs up with a weaker shadow effect. The numerical model of the shadow effect defines the shadow area, estimates the *δ* value within the shadow area to obtain the precursor molecules number, and finally predicts the nanostructures morphology behind the shading structures. The simulation results reveal that the surface diffusion effect is more prominent under the shadow effect, thus, the height difference of the processing structure is intensely reduced. The error between the simulation and experimental results is about 5%, confirming the reliability of the proposed model. To overcome the shadow effect, a relatively low beam current and short dwell time are recommended on initial growth, and recover to normal level to accelerate the additive process after reaching boundary height. Moreover, when fabricating groups of complex nanostructures, someone can refer to the numerical model to estimate the shadow area and decide whether it is necessary to turn the substrate around to face the nozzle halfway, considering it is troublesome to split the fabrication process especially when a bitmap or a STL file is used. The shading structure can reduce the precursor concentration behind it, and also can increase that in front of it. Therefore, someone can deliberately fabricate the shading structures to enhance the growth rate. The numerical model is irrelevant to the type of precursor gas and ion, so it can be extended to other precursor gases, such as W(CO)_6_, or electron beam induced deposition, to reproduce the shadow effect with different materials and processing parameters.

## Data Availability

Data are contained within the article or Supplementary Materials.

## Supplementary Materials

The following supporting information can be downloaded at: https://www.mdpi.com/article/10.3390/nano12060905/s1, Figure S1: The relationship between the shading length, boundary height and nozzle shift, the nozzle height, and the shading structure height. Table S1: The distances, dimensions, and growth rates of the pillars behind the rectangular shading struc-tures. Figure S2: Behind the shading structure. Figure S3: (a–e) The element map of the whole deposited nanostructures. (f–h) Element detection of the pillars behind shading structures [30,31,32]. Figure S4: The experimental results of the pillar arrays [30]. Figure S5. The δ distribution behind shading structures. Figure S6: The global simulation results in the CCA under the same fabrication parameters as the experiments. Table S2: The heights of experimental and simulation results with their errors and corresponding *δ* value.
