# Supplementary material for: Investigation of the Shadow Effect in Focused Ion Beam Induced Deposition
Source: Nanomaterials (Basel). 2022 Mar 9;12(6):905. doi: 10.3390/nano12060905 (PMC8955986; doi:10.3390/nano12060905)
Supplement: Supplementary file 1 [file nanomaterials-12-00905-s001.zip › nanomaterials-1611247-supplementary.pdf]

Supplementary Materials

Investigation of the Shadow Effect in Focused Ion Beam Induced Deposition

Chen Fang and Yan Xing *

Jiangsu Key Laboratory for Design and Manufacture of Micro-Nano Biomedical Instruments, Department of Mechanical Engineering, Southeast University, Nanjing, 211100, China; fangchen@seu.edu.cn
* Correspondence: xingyan@seu.edu.cn

Section S1. The Interactions between the Elements

Figure S1(a) demonstrates l and h_b as a function of L_{ns} when h = 100 µm, l_n = 5 µm and h_s = 5 µm. The shading structure features a rectangular appearance. If L_{ns} is below 100 µm, although the shadow effect occurs, h_b remains as 0. When the nozzle goes away, the shadow area will expand and cover the BIA position. The shading length is proportional to L_{ns}, with a coefficient h_s/h. Keeping the nozzle shift constant at 200 µm, l and h are in inverse proportion. When the nozzle is set close to the substrate (<200µm), the BIA position is covered by the shadow area. At this time, the boundary height has a linear function with the nozzle height and the slope is $-L_{ns}/l_n$, as shown in Figure S1(b). With the rise of the nozzle, the BIA position will not be affected by the shadow effect. The relationship between the shading length, the boundary height, and structure height can be explored by making the other parameters unchanged, as shown in Figure S1(c). The shading length is proportional to the structure height with a coefficient of L_{ns}/h. When the structure height is low (<2µm), the shadow area is too limited to cover the BIA position. The boundary height features a linear relationship with the structure height, and the slope is 1. The linear function has the intersection point $(0,-h\times l_n/L_{ns})$ on the y axis, which is the shading length caused by the height the same as the nozzle shift.

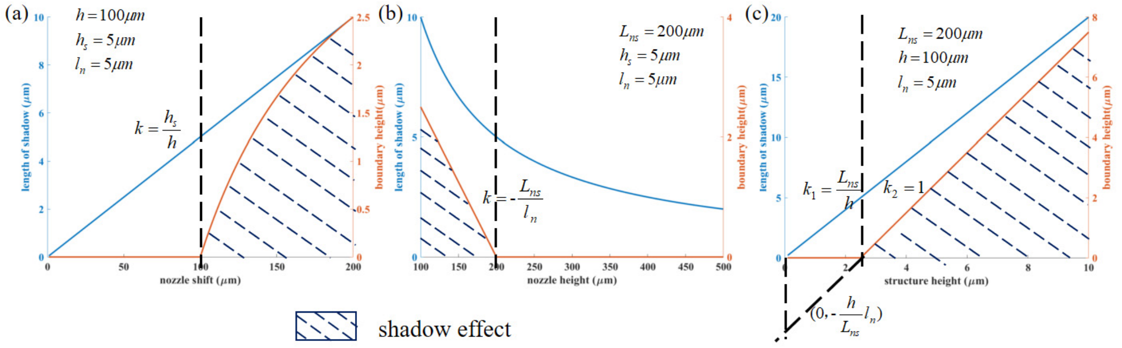

Figure S1. The relationship between the shading length, boundary height and nozzle shift, the nozzle height, and the shading structure height. (a) The shading length, boundary height as a function of the nozzle shift. (b) The shading length, boundary height as a function of the nozzle height. (c) The shading length, boundary height as a function of the structure height.

Table S1. The distances, dimensions, and growth rates of the pillars behind the rectangular shading structures. The precursor gas is phenanthrene, and the total processing time is 60 seconds.

	P_{as}	P_{a1}	P_{a2}	P_{a3}	P_{bs}	P_{b1}	P_{b2}	P_{b3}	P_{b4}
D(µm)	/	0.5	1	1.5	/	0.5	1	2.8	4.8
H(µm)	0.49	0	0.30	0.41	0.45	0	0.25	0.40	0.43
W(µm)	0.52	0.53	0.53	0.53	0.52	0.52	0.52	0.52	0.52
R(nm/s)	8.17	0	5.00	6.83	7.5	0	4.17	6.67	7.17

Section S2. The Influence of Shading Structures' Morphology

Figure S2(a) demonstrates the trend of the pillar heights with different positions, where center represents the midline, shift1μm represents the position shifting from the midline by 2 μm and 1 μm away from the structure, and shift4μm represents the position shifting from the midline by 2 μm and 4 μm away from the structure. To investigate the influence of the shadow effect on the composition of the deposition structure, we analyze the pillars by an energy dispersive X-ray spectrometer (EDS) to obtain the content of the pillar. The voltage is set as 15 kV. To verify the voltage is large enough to detect all the elements, we first scan the whole nanostructures. The detection results are demonstrated in Figure S3(a)~(e). All the elements can be detected under this voltage. Afterward, we select the single pillar to generate the element spectrum (spectrum 1, 2, 3), as shown in Figure S3(f)~(h). The pillars are mainly composed of C element, measuring up to 90 in %. In addition, there is a small amount of Ga element brought by the incident ions, and the other elements are almost negligible. It can be seen from Figure S2(b) and (c) that, when the height of the pillar is higher, the shadow effect goes weaker, increasing the concentration of precursor gas. Therefore, the content of C in the pillars also ascends, and the content of Ga declines. The mere exception is the pillar P_{e1}. In Figure 9(b), the P_{e1} is deformed due to the secondary effect during the fabrication of the pillar P_{e2} [1]. In the process of FIBID, sputtering coexists with deposition. The atoms sputtered from the P_{e2} strike at the surface of P_{e1}. The structure deposited by Ga ions with phenanthrene as precursor gas has a Ga core and a C shell [2]. Thus, the atoms sputtered from P_{e2} are mainly C atoms, contributing to the rise of the C element in P_{e1}. In addition, the amount of additional deposition is larger at the top of the P_{e1}. It is revealed that the sputtered atoms satisfy the spatial cosine distribution [3]. Therefore, the number of sputtered atoms received by the top of P_{e1} is the largest. The proximity effect does not happen behind the diamond and circular shading structures, which may be related to the gas concentration within the BIA and the precursor gas flow direction. Behind the rectangular shading structure, the gas flow is weaker compared with the other shading structures. Therefore, the sputter atoms have a lower possibility to be taken away.

However, the EDS measurements were made under 15 kV, which is a large voltage for the interested elements. Because of the large electron scattering, the shading structures also have a chance to generate C signal in the EDS spectrum, resulting in the increase of C concentration. More relevant research will be conducted to explain the phenomenon more deeply in the near future.

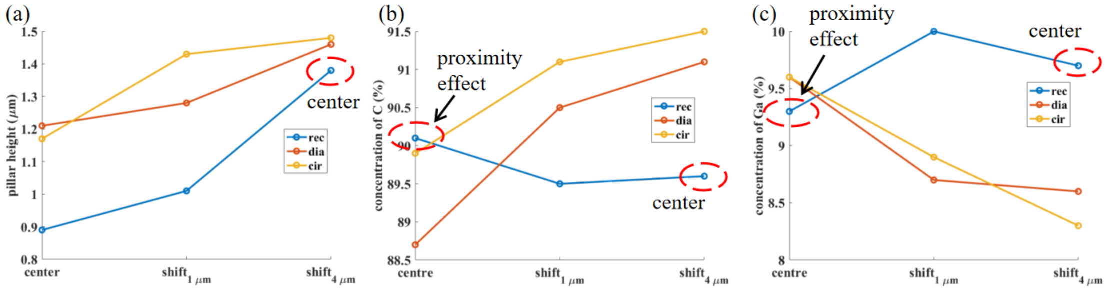

Figure S2. Behind the shading structure, (a) the height variation of the deposited pillars with different positions, (b) the variation of C content in the deposited pillars with different positions, and (c) the variation of Ga content in the deposited pillars with different positions.

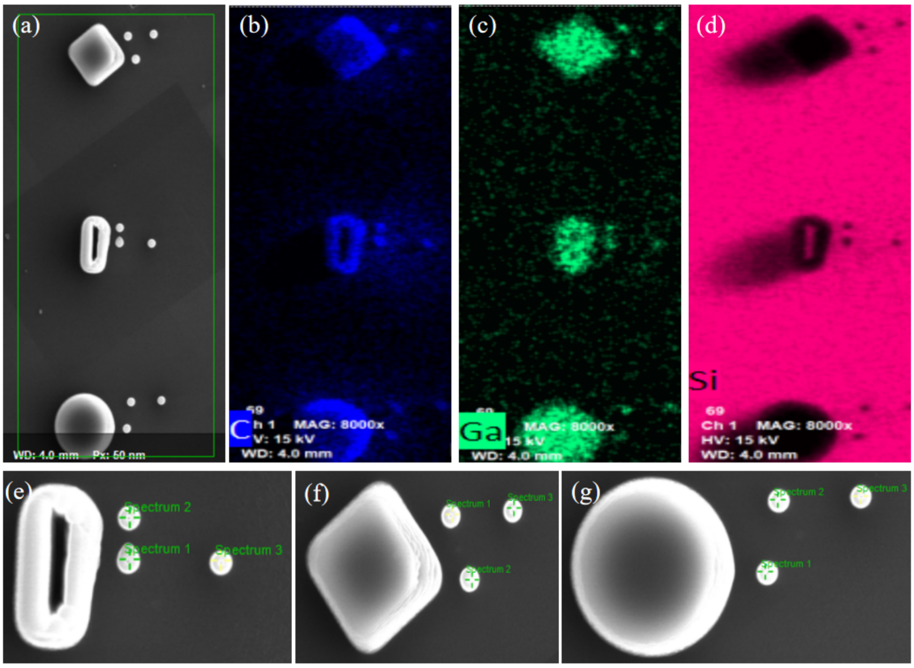

Figure S3. (a–e) The element map of the whole deposited nanostructures. (f–h) Element detection of the pillars behind shading structures.

Section S3. Experiments of the Pillar Arrays behind Shading Structures

In Figure S4, the pillar arrays are fabricated under the same beam parameters (90 pA, 30keV) with a fabrication time as 60 seconds for a single pillar. The spot mode is utilized for minimizing the precursor gas refreshment. It is noticed that, the secondary effect due to re-deposition is obvious. The pillars in the array except the last ones are coated with the re-deposition from their subsequent pillar fabrication process. The pillar height is not affected by the secondary effect. Without the shadow effect, the pillar can grow up to about 760 nm, and the curves of the pillar arrays converge to this value. The abnormal of the first pillar in C3 is due to the re-emitted precursor molecules from the shading structure [1].

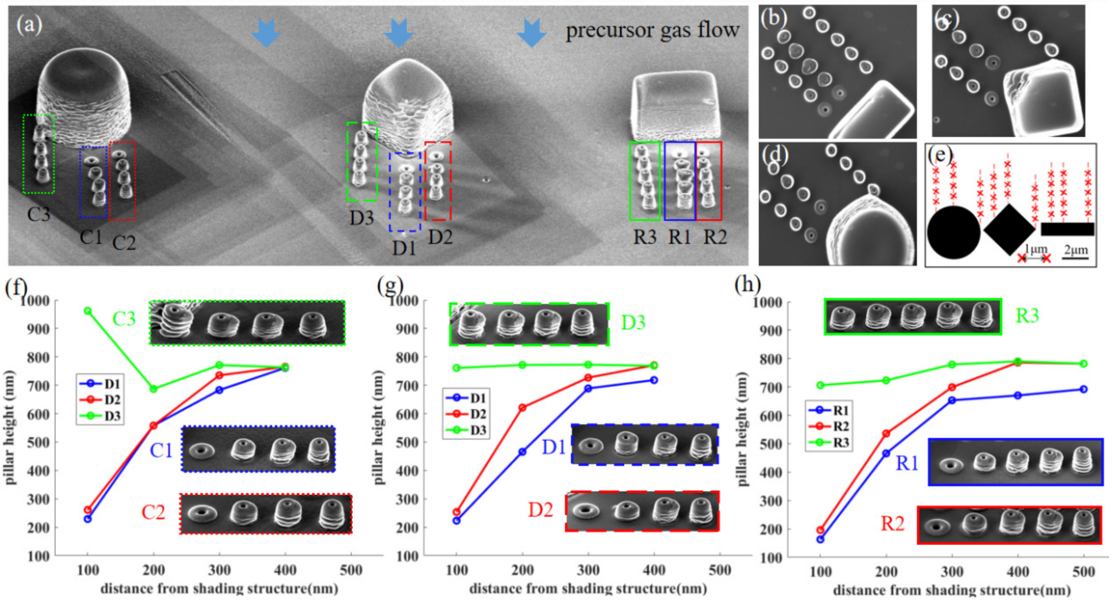

Figure S4. The experimental results of the pillar arrays. (a) The layout of the pillar arrays behind shading structures. (b–d) The top view of the pillar arrays. (e) The sketch of the pillar position. (f–h) The pillar height dependence on the distance from the shading structure.

Section S4. The Choice of Simulation Parameters

According to the SADM and PGFM, the ratio δ distribution is computed below in Figure S5. Three groups of simulation a~c are carried out behind three different shading structures: rectangular, diamond, and elliptical. The contour of the shading structure is spotted in a white dashed line. The rectangular shading structure is not marked, because the bottom edge of the picture represents the contour. The exponent q, which is an empirical parameter, is to tune the effect of shading structures characteristics. With a larger q, the shadow area features a 'slimmer waist' and a longer 'tail'. After several trial simulations, for group a~c, the shading length l is 10, 15, 15 μm, and the exponent q is 3, 3, 6. δ ranges from 0 ~ 1 behind all shading structures. With a longer shading length, the shadow area spreads to a larger scale, which brings a stronger shadow effect. The shadow effect behind the rectangular shading structure is the most prominent, and δ recovers slowly compared with the other two shading structures, as observed in our experiments. The elliptical, or like circular, shading structure imposes the weakest shadow effect because the precursor gas can easily flow along its surface to reach the shadow area. Moreover, behind the structure edge, δ recovers rapidly to the normal level, further stressing the importance of gas flow. The shadow effect in the horizontal direction parallel to the shading structures weakens, while strengthens in the vertical direction. In the experiments, the actual δ is also affected by the properties of precursor gas flow (e.g. velocity, temperature), which are difficult to detect and quantify. Therefore, the exponent q can be viewed as a fitting parameter to take the effects imposed by these properties into account.

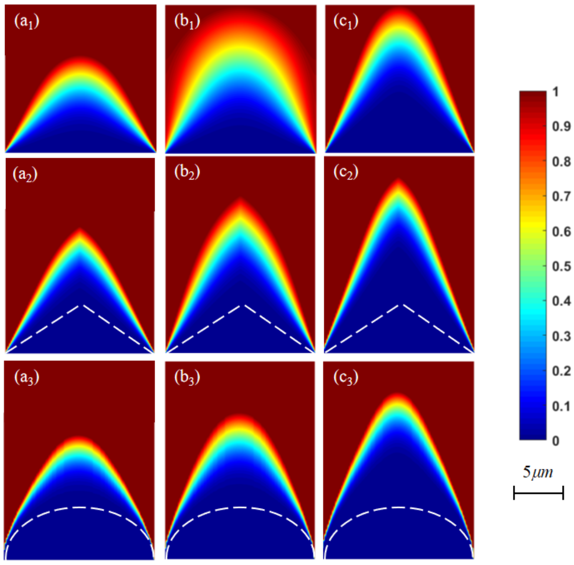

Figure S5. The δ distribution behind shading structures. Group a is emulated with a shading length of 10 μm and a q of 3. For groups b and c, the numbers are 15 μm, 15 μm, and 3, 6 respectively. The white dashed line refers to the shading structure contour.

Section S5. The global simulation of pillar growth

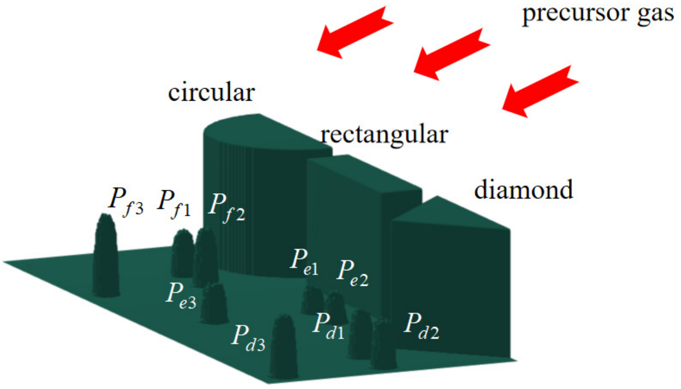

Figure S6. The global simulation results in the CCA under the same fabrication parameters as the experiments. The spacing between the shading structures is trimmed for accelerating simulation speed.

Table S2. The heights of experimental and simulation results with their errors and corresponding δ value.

	P_{e1}	P_{e2}	P_{e3}	P_{d1}	P_{d2}	P_{d3}	P_{f1}	P_{f2}	P_{f3}
δ	0.02	0.07	0.83	0.04	0.12	1	0.04	0.14	1
H_{exp}(μm)	0.89	1.01	1.38	1.21	1.28	1.46	1.17	1.43	1.48
H_{sim}(μm)	0.90	1.01	1.32	1.20	1.24	1.52	1.08	1.36	1.56
Error(%)	1.1	0	4.3	0.8	3.1	4.1	7.7	4.9	5.4

References

1. Fang, C.; Chai, Q.; Lin, X.; Xing, Y.; Zhou, Z. Experiments and simulation of the secondary effect during focused Ga ion beam induced deposition of adjacent nanostructures. *Mater. Des.* **2021**, *209*, 109993. doi:10.1016/j.matdes.2021.109993
2. Morita, T.; Kometani, R.; Watanabe, K.; Kanda, K.; Haruyama, Y.; Hoshino, T.; Kondo, K.; Kaito, T.; Ichihashi, T.; Matsui, S.; et al. Free-space-wiring fabrication in nano-space by focused-ion-beam chemical vapor deposition. *Vac. Sci. Technol. B Microelectron. Nanometer Struct.* **2003**, *21*, 2737. doi:10.1116/1.1630329
3. Chen, P.; Salemink, H.W.M.; Alkemade, P.F.A. Roles of secondary electrons and sputtered atoms in ion-beam-induced deposition. *J. Vac. Sci. Technol. B Microelectron. Nanometer Struct.* **2009**, *27*, 2718. doi:10.1116/1.3237147
